# Fentanyl-related overdose during incarceration: a comprehensive review

**DOI:** 10.1186/s40352-021-00138-6

**Published:** 2021-05-19

**Authors:** Eliana Kaplowitz, Ashley Q. Truong, Alexandria Macmadu, Meghan Peterson, Lauren Brinkley-Rubinstein, Nathan Potter, Traci C. Green, Jennifer G. Clarke, Josiah D. Rich

**Affiliations:** 1 The Center for Health and Justice Transformation, The Miriam Hospital, Providence, RI USA; 2 Center of Biomedical Research Excellence on Opioids and Overdose , The Rhode Island Hospital , RI Providence, USA; 3 Department of Mental Health, Johns Hopkins Bloomberg School of Public Health, Baltimore, MD USA; 4 Department of Epidemiology, Brown University School of Public Health, Providence, RI USA; 5 Division of General Internal Medicine, Albert Einstein College of Medicine, New York City, NY USA; 6 Department of Social Medicine, University of North Carolina, Chapel Hill, NC USA; 7 Christiana Care Health System, Newark, DE USA; 8 The Heller School of Social Policy and Management, Brandeis University, Waltham, MA USA; 9 Department of Emergency Medicine , Brown School of Medicine , RI Providence, USA; 10 Rhode Island Department of Health, Providence, RI USA

**Keywords:** Fentanyl, Opioid, Overdose, Incarceration

## Abstract

**Background:**

Fentanyl and related compounds have recently saturated the illicit drug supply in the United States, leading to unprecedented rates of fatal overdose. Individuals who are incarcerated are particularly vulnerable, as the burden of opioid use disorder is disproportionately higher in this population, and tolerance generally decreases during incarceration.

**Methods:**

We conduct a systematic search for publications about fentanyl overdoses during incarceration in PubMed and PsycINFO, as well as lay press articles in Google, from January 1, 2013 through March 30th, 2021.

**Results:**

Not a single fentanyl overdose was identified in the medical literature, but 90 overdose events, comprising of 76 fatal and 103 nonfatal fentanyl overdoses, were identified in the lay press. Among the 179 overdoses, 138 occurred in jails and 41 occurred in prisons, across the country.

**Conclusions:**

Fentanyl-related overdoses are occurring in correctional facilities with unknown but likely increasing frequency. In addition to the need for improved detection and reporting, immediate efforts to 1) increase understanding of the risks of fentanyl and how to prevent and treat overdose among correctional staff and residents, 2) ensure widespread prompt availability of naloxone and 3) expand the availability of medications to treat opioid use disorder for people who are incarcerated will save lives.

## Introduction

Opioid use and incarceration are interrelated problems in the United States. Even prior to the current opioid-driven overdose epidemic, up to one-third of people who used heroin were incarcerated annually (Boutwell, Nijhawan, Zaller, & Rich, 2007). Opioid use disorder (OUD) is more severe and advanced in justice-involved populations than in the general population (Winkelman, Chang, & Binswanger, 2018). While incarcerated, individuals often undergo a period of reduced opioid use that may lead to a diminished physiological tolerance to opioids (Binswanger et al., 2007). Reduced tolerance has been identified as a significant contributor to risk for drug overdose following release from incarceration, and it is estimated that individuals released from incarceration have nearly 13 times the risk of death as the general population, with overdose as the leading cause (Binswanger et al., 2016; Brinkley-Rubinstein et al., 2018; Green et al., 2018).

Fentanyl and related compounds have recently emerged as the primary drivers of the opioid overdose epidemic in the United States, having saturated much of the illicit drugs (Increases in Fentanyl Drug Confiscations and Fentanyl-Related Overdose Fatalities, 2018; O’Donnell, Halpin, Mattson, Goldberger, & Gladden, 2017; Ostling et al., 2018; Reported Law Enforcement Encounters Testing Positive for Fentanyl Increase Across US | Drug Overdose | CDC Injury Center, 2019; U.S. Drug Overdose Deaths Continue to Rise; Increase Fueled by Synthetic Opioids, 2019). As evidenced by toxicology reports and personal accounts from people who use illicit substances, illicit fentanyl is often mixed with heroin—although it has also recently been detected in cocaine and counterfeit opioid and benzodiazepine pills (Carroll, Marshall, Rich, & Green, 2017; Ciccarone, Ondocsin, & Mars, 2017; Korte, 2018; Suzuki & El-Haddad, 2017). Due to its lipophilic properties, fentanyl rapidly crosses the blood-brain barrier and binds to opioid receptors (Suzuki & El-Haddad, 2017). Estimated to be between 30 to 50 times more potent than heroin and 50 to 100 times more potent than morphine, fentanyl poses a much higher risk of overdose, even to those exposed to very small quantities (Ciccarone et al., 2017; Suzuki & El-Haddad, 2017).

In response to the worsening overdose crisis, policymakers and public health and medical institutions have launched initiatives to increase awareness of the risk and ubiquity of illicitly manufactured fentanyl in the community (U.S. Drug Overdose Deaths Continue to Rise; Increase Fueled by Synthetic Opioids, 2019). These efforts have primarily focused on promoting rigorously tested harm reduction measures, including education, naloxone distribution and access to low-barrier medication for opioid use disorder (MOUD) (Facing Addiction in America: The Surgeon General’s Spotlight on Opioids, 2019). Naloxone is a fast-acting opioid antagonist that blocks the effects of opioids and can thus reverse opioid overdose. While the number of MOUD programs increase, and access to naloxone continues to expand in the United States to include first responders, lay people, and individuals who are at risk for overdose in the community, to date these efforts have been limited in correctional facilities (Horton et al., 2017; Kim, Irwin, & Khoshnood, 2009; Wenger et al., 2019).

Correctional systems utilize intense surveillance, sanction and control to prevent illicit substances from entering into facilities (Kolind & Duke, 2016). Despite these efforts, nearly all correctional facilities have ongoing problems with contraband, ranging from cell phones to illicit substances (Kolind & Duke, 2016). Illicit fentanyl is inexpensive, widely available, and easy to smuggle into facilities to due to its high potency and need for only very small amounts (Increases in Fentanyl Drug Confiscations and Fentanyl-Related Overdose Fatalities, 2018; O’Donnell et al., 2017; Ostling et al., 2018; Reported Law Enforcement Encounters Testing Positive for Fentanyl Increase Across US | Drug Overdose | CDC Injury Center, 2019;U.S. Drug Overdose Deaths Continue to Rise; Increase Fueled by Synthetic Opioids, 2019). People who are incarcerated are at an increased risk of overdose when exposed to fentanyl, due to its high potency and the lower tolerance levels resulting from decreased opioid use while incarcerated.

### The present study

There are many causes of mortality in incarcerated populations including fatal drug overdose risk immediately following release, natural deaths, and intentional deaths during incarceration, including suicide on-site (Arfken, Suchanek, & Greenwald, 2017; Fazel & Benning, 2006; Kim et al., 2007; Larney, Topp, Indig, O’Driscoll, & Greenberg, 2012; Larney et al., 2014; Larney & Farrell, 2017; Mumola, n.d.; Salive, Smith, & Brewer, 1990). However, few studies have explored fentanyl-related overdose events in jails and prisons (Larney et al., 2014). Given the current fentanyl-driven overdose epidemic in the community and the presence of contraband drugs in correctional facilities, it is likely that there are fentanyl-related overdose deaths among individuals who are incarcerated. To better understand this, we conducted a comprehensive review of published reports of fentanyl-related overdose in correctional facilities, including jails and prisons. We aim to use our findings to gain a better understanding of the prevalence of fentanyl overdoses in prisons and jails in the United States to ultimately to reduce those overdoses and deaths.

## Methods

In this comprehensive review, we examined fentanyl-related overdose events during incarceration in a jail or prison. We defined a fentanyl-related overdose event as the occurrence of at least one fentanyl-related overdose on a given day at a correctional facility. Thus, one overdose event may involve multiple overdose victims. Fentanyl-related overdoses that occurred at the same correctional facility but on separate days were not defined as one overdose event; rather overdoses on each individual day comprised unique events. We defined a fentanyl-related overdose as a fatal or non-fatal overdose that was either confirmed or suspected to involve fentanyl, as stated by the source.

We systematically reviewed the medical literature for reports of fentanyl-related overdose events during incarceration from January 1, 2013 to March 30, 2021. PubMed and PsycINFO were used to identify reports in the medical literature. We reviewed articles published using the search terms: (“fentanyl overdose”) AND (prison OR jail).

Due to the fact that we found no cases in the peer-review literature, we expanded our search to include the lay press. Google search engine and Google alerts were used to identify cases of overdose in the media and lay press from January 1, 2013 to March 30, 2021. We used the same search terms, “fentanyl overdose in prison” and “fentanyl overdose in jail,” in the Google search engine. Our results yielded local and national news articles, blogs, and press releases. All results were manually reviewed and examined until we reached a full page of search results that contained no articles relevant to the review. For each reported fentanyl overdose event identified, an additional Google search was initiated using details of the event as search terms, such as the name of the correctional facility or the overdose victim. All relevant articles and details of the overdose events were cataloged and collated.

## Results

Not a single instance of fentanyl-related overdose reports or scientific articles among incarcerated individuals was identified through PubMed or PsycINFO. Our media analysis and search of articles in the lay press returned 113 relevant reports. After duplicate reports were combined, our results identified a total of 90 reported events comprising 179 fentanyl-related overdoses during the study period. Of these 179 fentanyl-related overdoses, 76 were fatal and 103 were nonfatal. One victim overdosed twice on different days (Hopkins, 2017). All United States regions were represented; specifically, reported overdose events occurred across 32 states and the District of Columbia (DC). California, Florida, Pennsylvania and Ohio were overrepresented with a total of 40, 18, 11, and 10 overdoses, respectively. Of note, 13 of the 40 overdoses in California resulted from a single events at the Mule Creek State Prison in April 2018 (Goldberg, 2018). Based on the U.S. Department of Agriculture’s Rural-Urban classification scheme (USDA ERS - Rural-Urban Continuum Codes*,* n.d.), 72 overdose events occurred in metropolitan counties, 17 events occurred in nonmetropolitan countries with urban populations, and 1 event occurred in Washington, DC. Characteristics of the known and suspected fentanyl overdose cases among incarcerated individuals are presented in Table 1.

**Table 1:** Date, location, circumstances of fentanyl-related overdose incidents, United States, 2013-March 2021

Date	State	Facility	Fatal	Nonfatal	Fentanyl Confirmed	Mode of Entry	Naloxone
2013 (Fairbanks, 2016)	NY	Attica Correctional Facility	3	0	Yes	Yes	No
May 2014 (McKelway, 2015)	VA	Henrico County Jail	1	0	Yes	Yes	No
May 2014 (Doug Rogers, 2015)	PA	Erie County Prison	1	3	Yes	Yes	No
May 2015 (Woman died of fentanyl overdose at Cook County Jail: Autopsy, 2019)	OR	Multnomah County Detention Center (Justice Center)	0	3	Yes	Yes	No
June 2015(Mitchell, 2018)	CO	Gunnison County Jail	1	0	Yes	No	No
July 2015 (Denson, n.d.)	ME	York County Jail	0	3	No	No	No
Oct 2015 (Sturgeon, 2015)	NJ	Bayside State Prison	0	1	Yes	Yes	Yes
Mar 2016 (Sandy, 2016)	OH	Lorain County Jail	1	0	Yes	No	No
Mar 2016 (Corwin, 2016)	NH	Hillsborough County Department of Corrections	1	0	Yes	No	No
Apr 2016 (Dolan, n.d.)	ME	Cumberland County Jail	1	0	Yes	No	No
Sept 2016 (Crittenden Woman Sentenced to 224 Months for Distribution of Drugs Resulting in Death, 2016)	KY	Kenton County Jail	1	0	Yes	Yes	No
Nov 2016 (Schaefer, 2016)	OH	Montgomery County Jail	1	0	Yes	Yes	No
Dec 2016 (Treleven, 2020)	WI	Dane County Jail	1	0	Yes	No	No
Jan 2017 (Berg, 2019)	MI	Lakeland Correctional Facility	1	2	No	Yes	No
May 2017 (Harlow, 2017)	ME	Somerset County Jail	0	1	No	No	Yes
May 2017 (Alexander, 2017)	D.C.	D.C. Jails	2	0	Yes	No	No
July 2017 (Loannou, 2017)	CA	Santa Clara County Main Jail	0	4	No	No	No
July 2017 (Coroner: LaPorte County Jail Inmate Died by Accidental Overdose, 2017)	IN	LaPorte County Jail	1	0	Yes	No	No
Oct 2017 (Burger, 2017)	OH	Franklin County Jail	1	0	Yes	No	No
Oct 2017 (Hopkins, 2017)	AK	Hiland Mountain Correctional Center	0	5	Yes	Yes	Yes
Nov 2017 (Craig, 2019)	NY	Livingston County Jail	1	0	Yes	Yes	No
Nov 2017 (Ward, n.d.)	KY	Montgomery County Regional Jail	1	0	Yes	Yes	No
Dec 2017(Ovalle, 2017)	FL	Miami-Dade Jail	2	0	No	No	No
Jan 2018 (Naylor, 2018)	PA	Cumberland County Prison	1	0	Yes	Yes	No
Feb 2018 (Bustos, 2018)	FL	Venice Police Department	1	0	Yes	No	No
Apr 2018 (Stucker, 2019)	NH	Strafford County House of Corrections	1	0	Yes	Yes	No
Apr 2018 (Mitchell, 2018)	TN	Sumner County Jail	0	6	No	Yes	No
Apr 2018 (Goldberg, 2018)	CA	Mule Creek State Prison	1	12	Yes	No	No
May 2018 (Burns, 2018)	NC	Durham County Jail	1	0	Yes	No	No
May 2018 (Broden, 2018)	TN	Rutherford County Jail	0	1	Yes	Yes	Yes
June 2018(Bogel-Burroughs, 2018)	MD	Baltimore Central Booking and Intake Center	1	0	Yes	No	No
June 2018 (Jane Wester & Kane, 2018)	NC	Mecklenburg County Jail	1	0	Yes	No	No
July 2018 (Wester, n.d.)	NC	Mecklenburg County Jail	1	0	Yes	No	No
Aug 2018(Lisenby, 2018)	MO	St. Louis Medium Security Institution	1	0	Yes	No	No
Aug 2018 (Brookbank, 2019)	OH	Ross Correctional Institution	0	2	Yes	No	No
Sept 2018 (Miller, 2018)^R^	FL	Marion County Jail	1	0	Yes	Yes	No
Nov 2018 (Signorini, 2019)	PA	Allegheny County Jail	1	0	Yes	No	No
Nov 2018 (Perlstein, 2019)	LA	Orleans Justice Center	1	0	Yes	No	No
Dec 2018^D^(Drug may be to blame for Albany County inmate’s death*,* n.d.)	NY	Albany County Jail	1	0	Yes	Yes	No
Dec 2018 (Klein, 2018)	KY	Boyd County Jail	2	0	No	Yes	No
Dec 2018 (Sutyak, 2018)	OH	Portage County Jail	0	1	Yes	No	No
Dec 2018 (Murphy, n.d.)	NY	Nassau County Jail	1	0	No	No	No
Jan 2019 (Jail Sweep Underway after Pasco Inmate Dies from Drug Overdose, 2019)	FL	Land O′ Lakes Detention Center	1	4	Yes	Yes	Yes
Jan 2019 (Pasco Man Pleads Guilty To Causing Series Of Overdoses While Incarcerated At The Pasco County Jail, 2019)	FL	Pasco County Jail	0	3	Yes	Yes	Yes
Jan 2019 (Deike, 2019)	OH	Cuyahoga County Jail	0	1	Yes	No	No
Feb 2019 (Pasco County man responsible for numerous overdoses at Pasco County jail sentenced to 27 years in federal prison*,* 2020)	FL	Pasco County Jail	1	1	Yes	Yes	Yes
Mar 2019 (Woman died of fentanyl overdose at Cook County Jail: Autopsy*,* 2019)	IL	Cook County Jail	1	0	Yes	No	No
Mar 2019 (WKBN Staff, 2019)	OH	Mahoning County Jail	0	1	Yes	No	Yes
Mar 2019 (Rains, 2019)	AR	Sebastian County Detention Center	1	0	Yes	No	No
Mar 2019 (Schwigert, 2019)	PA	Lancaster County Prison	1	0	Yes	Yes	No
Apr 2019 (Owens, 2019)	CT	Hartford Correctional Center	1	3	Yes	No	Yes
Apr 2019 (J Wester, 2019)	NC	Mecklenburg County Jail	1	0	Yes	No	No
Apr 2019 (3 Inmates Connected to Jail Overdoses, 2019)	AZ	Mohave County Jail	0	5	Yes	Yes	No
Apr 2019 (Goodrich, 2019)	MN	Blue Earth County Jail	0	1	Yes	Yes	No
May 2019 (City News Services, 2019)	CA	Theo Lace Jail	1	0	Yes	No	No
May 2019 (Dave Rogers, 2019)	MA	Middleton Jail	1	0	Yes	No	No
May 2019 (Robert Patrick, 2019; Roberts Patrick, 2020)	MO	St. Charles County Jail	1	0	No	No	No
June 2019 (Smay, 2019)	WA	Spokane County Jail	1	0	Yes	No	No
July 2019 (Berstein, 2020)	OR	Multnomah County Jail	1	0	Yes	Yes	Yes
July 2019 (City News Services, 2019)	CA	San Diego Central Jail	1	0	Yes	No	No
Aug 2019 (Ferrise, 2019)	OH	Cuyahoga County Jail	1	0	Yes	No	No
Aug 2019 (Associated Press, 2019b)	MO	St. Louis Medium Security Institution	1	0	Yes	No	Yes
Sept 2019 (Harris, 2019)	FL	Columbia Correctional Institution	2	1	No	No	No
Oct 2019 (Barbra, 2019)	CA	San Francisco County Jail	0	5	No	No	Yes
Oct 2019 (Poli, n.d.)	OH	Franklin County Jail	1	0	Yes	No	No
Oct 2019 (Associated Press, 2019a)	MO	Moberly Correctional Center	2	0	Yes	No	No
Nov 2019 (Dickson, n.d.)	MI	Women’s Huron Valley Correctional Facility	1	0	Yes	No	Yes
Jan 2020 (McDonald, 2020)	CA	San Diego Central Jail	1	0	Yes	No	No
Feb 2020 (Schmidt, 2020)	IL	Madison County Jail	0	3	Yes	Yes	No
Feb 2020 (Associated Press, 2020a)	PA	Curran-Fromhold Correctional Facility	3	1	Yes	No	No
Mar 2020 (Winston, 2020)	FL	Palm Beach County Jail	1	0	Yes	No	No
Apr 2020 (Lubinski, 2020)	MO	Clay County Detention Center	1	6	No	No	Yes
May 2020 (Camden County Jail Inmate Admits Providing Drugs That Caused Fellow Inmate’s Overdose Death, 2021)	GA	Camden County Detention Facility	1	0	Yes	Yes	No
June 2020 (Finn, 2020)	NY	Yaphank Facility	0	1	No	No	No
June 2020 (Finn, 2020)	NY	Riverhead Jail	0	5	No	No	No
June 2020 (Sledge, 2020)	LA	New Orleans Jail	1	0	Yes	No	No
July 2020 (Craig, 2020)	MI	Michigan Department of Corrections	1	0	No	No	No
Aug 2020 (Coroner: Overdose Caused Iroquois Co. Inmate’s Death, 2020; Iroquois County Inmate Died of Fentanyl, Heroin Overdose, 2020)	IL	Iroquois County Jail	2	0	Yes	No	No
Aug 2020 (Gartrell, 2020)	CA	Contra Costa Jail	1	0	Yes	No	No
Aug 2020 (Hlavaty, 2021))	OH	Cuyahoga County Jail	1	0	Yes	Yes	No
Sep 2020 (Minneapolis Man Charged With Smuggling Heroin Into Hennepin County Jail*,* n.d.; Nelson, 2020, p. 3)	MN	Hennepin County Jail	0	3	No	Yes	Yes
Sep 2020 (Associated Press, 2020b; Schafer, 2020)	WI	Manitowoc County Jail	1	0	Yes	Yes	No
Oct 2020 (Ireland, 2020)	CA	Vista Detention Facility	1	0	Yes	No	No
Nov 2020 (Ireland, 2021; Riggins, 2021)	CA	Otay Mesa Jail	1	0	Yes	No	No
Dec 2020 (WDTV News Staff, 2020)	WV	North Central Regional Jail	2	1	No	No	No
Dec 2020 (Saunders, 2020)	CA	San Diego Central Jail	0	6	No	No	Yes
Dec 2020 (Kemp, n.d.; Lee, 2020)	CA	Mendocino County Jail	0	2	No	No	Yes
Jan 2021 (Nuttle, 2021; Stanton, 2021)	CA	Sacramento County Jail	0	4	No	Yes	Yes
Feb 2021 (Gottlieb, 2021)	WA	Clallam County Correctional Facility	0	1	Yes	Yes	Yes
Mar 2021 (Sheriff’s Office Conducts Drug Search in Jail After Drug Overdose*,* n.d.)	NC	Bladen County Detention Facility	0	1	No	No	No

**Fig. 1:**
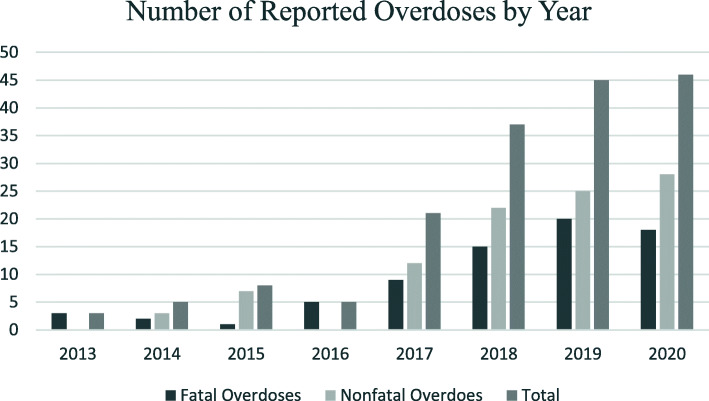
Number of reported overdoses by year (2013–2020)

Table 2 presents the breakdown of fatal and non-fatal overdoses in prisons and jails, and Fig. 1 presents overdoses in correctional settings over time.

**Table 2:** Number of reported fatal and nonfatal overdoses by facility type (prison or jail)

	Fatal Overdoses	Nonfatal Overdoes
Prison	15	26
Jail	61	77
Total	76	103

### Confirmation of fentanyl

In 77% (*n* = 70) of included events, the involvement of fentanyl or its analogs was reported by the source. However, only a few overdose events (*n* = 12) were reported to include a systematic toxicology screening upon overdose. Most articles did not specify whether exposure to fentanyl was intentional or unintentional. During an overdose event in Covington, Kentucky on September 4, 2016, a young woman at the Kenton County Jail unintentionally consumed fentanyl and died of a fatal overdose (Crittenden Woman Sentenced to 224 Months for Distribution of Drugs Resulting in Death*,* 2016). Toxicology reports revealed a mixture of fentanyl and morphine in her body at the time of death; however, both her mother—who delivered the substance through two individuals who were incarcerated at the jail—and the individual her mother purchased the substance from believed it to be heroin (Crittenden Woman Sentenced to 224 Months for Distribution of Drugs Resulting in Death*,* 2016).

### Fentanyl entrance

In outlining the circumstances around the fentanyl-related overdose events, 33 (37%) reports also described how fentanyl entered the correctional facility. Purported modes of entry included being smuggled in by a family member, a food service employee, a visitor during an open house, and an individual who was incarcerated and hid a bag of fentanyl inside her body. Many of the reports in the lay press emphasized the tight surveillance and security measures within correctional settings.

### Response to overdose

Naloxone was administered to overdose victims in 20 (22%) events, one of which occurred in 2015 and the remainder of which occurred between 2017 and 2021. While naloxone was administered either by correctional or medical staff, individuals who were incarcerated played a critical role in response to overdoses in a couple of instances, such as when they notified correctional staff at the Lorain County Jail in Elyria, Ohio about an individual who was incarcerated who was overdosing (Sandy, 2016). In another instance from May 2014, individuals who were incarcerated at the Henrico County Jail in Henrico, Virginia applied ice on parts of the victim’s body in attempts to revive him after he overdosed, which is an ineffective method to reverse opioid overdose (McKelway, 2015). The overdose accounts included in this review demonstrate how the onset of fentanyl-related overdose symptoms is rapid. In an article describing a fatal overdose at the Baltimore Central Booking and Intake Center in June 2018, a victim’s health is described as having deteriorated rapidly after ingesting what was later confirmed to be a mixture of morphine and fentanyl. Less than an hour prior to overdosing, he was described as “cool, calm, relaxed” (Bogel-Burroughs, 2018). In another fatal overdose case, an individual who was incarcerated died on June 28, 2015 while in police custody at the Gunnison County Jail in Gunnison, Colorado. A check performed at 9:01 am did not raise concerns about his health. However, he was found unconscious 3 minutes later (Michell, 2016).

## Discussion

This is the first paper to summarize fentanyl-related overdose events that occurred during incarceration. No reports were identified in the medical literature. However, we identified 90 overdose events totaling 179 overdoses in the lay press and these reports are rapidly increasing. The large discrepancies in reporting between the lay press and the medical literature suggests under-reporting of overdose events in prisons and jails, and a lack of attention given to the opioid crisis in correctional settings more generally. The number of fentanyl overdoses is likely much higher, as in an article published in the Orange County Register, a sheriff reported that 129 doses of naloxone had been administered to 70 individuals in 2019 (Saavedra, 2020), and yet, our media search revealed only a single reported overdose event in Orange County that year (OC Inmate’s Death Caused By Fentanyl Overdose, Officials Say, 2020). This suggests a vast under reporting of overdoses, and likely fentanyl overdoses in prisons and jails. We suspect that correctional administrators endure tremendous pressure to minimize the prevalence of substances and contraband inside, in an effort to maintain the perception that jails are safe and rehabilitative. For example, our team is anecdotally aware of six events of fentanyl related overdoses and deaths, that occurred in correctional facilities in two different states where the attending clinicians were told by their superiors not to report these cases in the medical literature.

As fentanyl contamination of the drug supply continues in the community, we expect its presence in correctional facilities to likewise rise in parallel. However, the scope of the problem is unclear because fentanyl overdoses in correctional facilities are not being systematically tracked. Unfortunately, as demonstrated by the few reports of systematic toxicology screening in our study, there currently is no standard of care for evaluating deaths among people who were incarcerated; ideally, an autopsy and complete toxicologic screening should be completed for all deaths occurring in correctional facilities. Enforcing a standard of care for all deaths and a system to systematically track deaths in correctional settings is essential to fully understanding the scope of the crisis.

However, further data is not necessary to begin addressing overdoses and deaths in corrections. Three evidence-based interventions can, and must, be implemented 1) educate correctional staff and individuals who are incarcerated about the risks of fentanyl, 2) ensure widespread, readily available naloxone accompanied with training on how to properly administer it, and 3) expand availability of medications for opioid use disorder in prisons and jails.

Our study found that naloxone was reported to be administered in only 22% of included overdose events. Both correctional staff and individuals who are incarcerated should be educated on the growing prominence of fentanyl, its effects, signs of an opioid overdose, and the use of naloxone. As a harm reduction measure, naloxone trainings should be widely available to all staff and individuals who are incarcerated. Studies of naloxone training in the correctional setting underscore the potential for individuals who are incarcerated to participate in trainings and correctly administer intranasal naloxone while incarcerated (Green, Ray, Bowman, McKenzie, & Rich, 2014; Kobayashi et al., 2017; Parmar, Strang, Choo, Meade, & Bird, 2017; Petterson & Madah-Amiri, 2017). At a minimum, naloxone should be present in all correctional units, and units should be staffed with correctional officers who are trained in naloxone administration. As of November 2018, RIDOC implemented the first statewide naloxone training for correctional officers, which included trainings to recognize overdose symptoms and correctly administer naloxone. Naloxone is now offered to RIDOC officers who completed the training and is readily available in all Rhode Island prisons (personal communication Jennifer Clarke, MD, MPH).

In addition to naloxone distribution, MOUD remain the gold-standard of treatment for OUD and an effective harm-reduction intervention to prevent overdose (Facing Addiction in America: The Surgeon General’s Spotlight on Opioids, 2019). The availability of and attitudes toward MOUD in correctional facilities are rapidly changing given the magnitude of the epidemic and the demonstrated decrease in overdose deaths among individuals recently released from incarceration. In a randomized clinical trial conducted by Kinlock and Gordon et al. in Baltimore, Maryland, participants who were incarcerated and assigned to receive both counseling and methadone were less likely to test positive for opioids at one-, three-, six-, and twelve-month follow-ups in the community compared to those who received only counseling (Kinlock et al., 2007; Kinlock, Gordon, Schwartz, Fitzgerald, & O’Grady, 2009; Kinlock, Gordon, Schwartz, & O’Grady, 2008). Our team has also demonstrated that individuals who are maintained on methadone while incarcerated are more likely to return to their methadone clinic after they are released than those who undergo forced withdrawal while incarcerated (Rich et al., 2015). Thus, not only does providing access to MOUD in corrections increase individual’s chance of recovery upon reentry, it also reduces risk of overdose during and after incarceration. Despite the robust literature demonstrating its efficacy, the use of MOUD in correctional facilities is still met with resistance. According to the Jail and Prison Opioid Project (JPOP), only 356 correctional facilities out of the nearly 5000 facilities in the United States offer any form of MOUD to individual who are incarcerated (Prison Opioid Project – Medication for Opioid Use Disorder and the Criminal Justice System, n.d.).The compounded problem of extremely concentrated, widely available and affordable fentanyl, and the lowered tolerance of individuals during incarceration, necessitates evidence-based interventions. Generally, there has been a lack of response among correctional facilities to address the growing opioid crisis; our study found that among the facilities that have attempted to respond to the overdose crisis, they have predominantly done so through increasing security and surveillance. However, a recent study found that increased surveillance and punishment among people using opioids was correlated to less likelihood to seek treatment and begin recovery (Mazhnaya et al., 2016). Harm-reduction interventions in the community have effectively reduced risk of overdose; however, there has been little focus on promoting these in criminal justice settings as well.

Our findings should be interpreted in light of several limitations. To our knowledge, no scientific papers about fentanyl overdose during incarceration have been published, and cases reported in the lay press likely represent only a fraction of all overdose events. Moreover, due to the 2019 novel coronavirus (COVID-19) pandemic, media coverage on correctional facilities has likely shifted towards COVID-19 outbreaks in facilities and away from fentanyl-related overdoses. Reluctance to release data on overdose within corrections likely also results in the underreporting of fentanyl-related overdoses. More systematic data collection, reporting, and surveillance of unintentional drug-induced deaths is needed to understand the magnitude of fatal and nonfatal fentanyl-related overdoses in the incarcerated population.

## Conclusions

Fentanyl-related overdoses in correctional facilities are likely spill-over from the opioid epidemic in the community. Fear and misinformation around fentanyl exposure have contributed to the stigmatization of OUD and resistance towards effective harm reduction measures and treatment. Illicit drug use in correctional facilities occurs regularly. In the face of this evolving fentanyl-driven opioid epidemic, correctional staff, individuals who are incarcerated, and the public must be equipped with the knowledge and tools necessary to reduce and prevent fatal and nonfatal overdose events. Naloxone and MOUD, specifically, are beneficial after release and should also be safe and effective in jails and prisons. Moreover, systematic data collection, including toxicology screens, and increased reporting of fatalities in corrections are urgently needed to monitor and curb the opioid overdose epidemic.

## References

[CR1] *3 inmates connected to jail overdoses*. (2019, May 15). Kingman Daily Miner. https://kdminer.com/news/2019/may/15/3-inmates-connected-jail-overdoses/

[CR2] Alexander, K. L. (2017, June 20). Two D.C. jail inmates found dead in May died from opioid overdoses. Washington Post. https://www.washingtonpost.com/local/public-safety/two-dc-jail-inmates-found-dead-in-may-died-from-opioid-overdoses/2017/06/20/b2085796-55fa-11e7-a204-ad706461fa4f_story.html

[CR3] Arfken CL, Suchanek J, Greenwald MK (2017). Characterizing fentanyl use in methadone-maintained clients. Journal of Substance Abuse Treatment.

[CR4] Associated Press. (2019a). *Autopsy: 2 Moberly Inmates Died From Overdose Deaths*. US News & World Report. //www.usnews.com/news/best-states/missouri/articles/2019-12-05/autopsy-2-moberly-inmates-died-from-overdose-deaths

[CR5] Associated Press. (2019b). Inmate’s Death at St*.* Louis Jail Blamed on Fentanyl Overdose. US News & World Report. https://www.usnews.com/news/best-states/missouri/articles/2019-10-18/inmates-death-at-st-louis-jail-blamed-on-fentanyl-overdose

[CR6] Associated Press. (2020a, March 5). *3 inmates in Philadelphia die of fentanyl overdose; jail investigating* [Text.Article]. Fox29; FOX 29 news Philadelphia. https://www.fox29.com/news/3-inmates-in-philadelphia-die-of-fentanyl-overdose-jail-investigating

[CR7] Associated Press. (2020b, December 2). Manitowoc co*.* Jail inmate dies of fentanyl overdose, charges recommended. https://www.nbc15.com/2020/12/02/manitowoc-co-jail-inmate-dies-of-fentanyl-overdose-charges-recommended/

[CR8] Barbra, M. (2019, October 8). Fentanyl scare sends 9 deputies, 5 inmates to hospital. The San Francisco Examiner. https://www.sfexaminer.com/news/narcotic-exposure-at-sf-county-jail-sends-5-deputies-5-inmates-to-hospital/

[CR9] Berg, K. (2019, October 9). Lawsuit*: Charlotte man died of overdose from prison drug smuggling ring*. Lansing State Journal. https://www.lansingstatejournal.com/story/news/2019/12/09/lawsuit-charlotte-man-died-overdose-prison-drug-smuggling-ring/4351994002/

[CR10] Berstein, M. (2020, September 30). $10 million lawsuit filed against Multnomah County in 2019 drug overdose death of inmate. Oregonlive. https://www.oregonlive.com/crime/2020/09/10-million-civil-rights-suit-filed-against-multnomah-county-stemming-from-2019-death-of-inmate-who-died-from-drug-overdose-in-custody.html

[CR11] Binswanger IA, Stern MF, Deyo RA, Heagerty PJ, Cheadle A, Elmore JG, Koepsell TD (2007). Release from prison—A high risk of death for former inmates. New England Journal of Medicine.

[CR12] Binswanger IA, Stern MF, Yamashita TE, Mueller SR, Baggett TP, Blatchford PJ (2016). Clinical risk factors for death after release from prison in Washington state: A nested case-control study. Addiction (Abingdon, England).

[CR13] Bogel-Burroughs, N. (2018, August 8). Disabled man who died in Baltimore jail overdosed on morphine and fentanyl; advocates ask why he was there. The Baltimore Sun. https://www.baltimoresun.com/news/crime/bs-md-ci-jail-death-autopsy-20180808-story.html

[CR14] Boutwell AE, Nijhawan A, Zaller N, Rich JD (2007). Arrested on heroin: A national opportunity. Journal of Opioid Management.

[CR15] Brinkley-Rubinstein L, Macmadu A, Marshall BDL, Heise A, Ranapurwala SI, Rich JD, Green TC (2018). Risk of fentanyl-involved overdose among those with past year incarceration: Findings from a recent outbreak in 2014 and 2015. Drug and Alcohol Dependence.

[CR16] Broden, S. (2018). Inmate overdoses while Rutherford County jail waits on contraband scanner arrival. The Daily News Journal. https://www.dnj.com/story/news/2018/07/09/inmate-overdose-sparks-rutherford-sheriff-seek-scanner-detect-drug-smuggling/767558002/

[CR17] Brookbank, S. (2019, August 30). *Ross overdose: Heroin-fentanyl mix sickened 29 at prison*. https://www.cincinnati.com/story/news/2018/08/30/ross-overdose-heroin-fentanyl-mix-sickened-29-prison/1142138002/

[CR18] Burger, B. (2017, December 8). Coroner determines jail inmate died of fentanyl overdose. The Columbus Dispatch. https://www.dispatch.com/news/20171208/coroner-determines-jail-inmate-died-of-fentanyl-overdose

[CR19] Burns, M. (2018, July 19). *Autopsy: Durham inmate died of drug overdose :* WRAL.Com. https://www.wral.com/autopsy-durham-inmate-died-of-drug-overdose/17708319/

[CR20] Bustos, J. (2018, February 22). Man dies while in custody of Venice IL police | Belleville news-democrat. Belleville News-Democrat. https://www.bnd.com/news/local/article201620669.html

[CR21] *Camden County jail inmate admits providing drugs that caused fellow inmate’s overdose death*. (2021, March 2). The United States Department of Justice, U.S. Attorney’s Office. https://www.justice.gov/usao-sdga/pr/camden-county-jail-inmate-admits-providing-drugs-caused-fellow-inmates-overdose-death

[CR22] Carroll JJ, Marshall BDL, Rich JD, Green TC (2017). Exposure to fentanyl-contaminated heroin and overdose risk among illicit opioid users in Rhode Island: A mixed methods study. International Journal of Drug Policy.

[CR23] Ciccarone D, Ondocsin J, Mars SG (2017). Heroin uncertainties: Exploring users’ perceptions of fentanyl-adulterated and -substituted ‘heroin’. International Journal of Drug Policy.

[CR24] City News Services. (2019, October 29). *2 jail deaths blamed on drug overdoses*. FOX 5 San Diego. https://fox5sandiego.com/news/2-jail-deaths-blamed-on-drug-overdoses/

[CR25] Coroner: LaPorte County Jail inmate died by accidental overdose. (2017, August 9). https://www.wndu.com. https://www.wndu.com/content/news/Coroner-LaPorte-County-Jail-inmate-died-by-accidental-overdose-439469063.html

[CR26] *Coroner: Overdose caused Iroquois Co. Inmate’s death*. (2020, September 29). WAND-TV. https://www.wandtv.com/news/coroner-overdose-caused-iroquois-co-inmates-death/article_36033fda-0290-11eb-bb09-27d2bd23d048.html

[CR27] Corwin, E. (2016, April 20). Jailed homeless activist died of Fentanyl Overdose*,* Says Medical Examiner. NHPR. https://www.nhpr.org/post/jailed-homeless-activist-died-fentanyl-overdose-says-medical-examiner

[CR28] Craig. (2020, July 24). MSP investigating prison overdose death during ban on visitors due to coronavirus. WXYZ. https://www.wxyz.com/news/msp-investigating-prison-overdose-death-during-ban-on-visitors-due-to-coronavirus

[CR29] Craig, G. (2019, February 8). Livingston County Sheriff’s deputies sued over death of man who overdosed in jail. Democrat and Chronicle. https://www.democratandchronicle.com/story/news/2019/02/08/livingston-county-sheriffs-deputies-sued-over-jail-overdose-death/2800291002/

[CR30] *Crittenden Woman Sentenced to 224 Months for Distribution of Drugs Resulting in Death*. (2016, August 11). United States Department of Justice. https://www.justice.gov/usao-edky/pr/crittenden-woman-sentenced-224-months-distribution-drugs-resulting-death

[CR31] Deike, J. (2019, January 23). *Nurse attacked, narcotics seized at Cuyahoga County Jail as crisis deepens*. https://www.cleveland19.com. https://www.cleveland19.com/2019/01/23/nurse-strangled-narcotics-seized-cuyahoga-county-jail-crisis-deepens/

[CR32] Denson, B. (n.d.). 3 OD on dangerous drug in Portland jail; feds say inmate smuggled contraband inside her body. The Oregonian. Retrieved April 12, 2021, from https://www.oregonlive.com/portland/2015/03/three_od_on_dangerous_drug_smu.html

[CR33] Dickson, J. D. (n.d.). November death of Michigan inmate, 37, was fentanyl overdose. The Detroit news. Retrieved March 16, 2021, from https://www.detroitnews.com/story/news/local/michigan/2020/03/03/november-death-michigan-inmate-37-fentanyl-overdose/4940142002/

[CR34] Dolan, S. (n.d.). *Portland jail inmate’s drug death classified as accidental*. Press Herald Retrieved April 12, 2021, from https://www.pressherald.com/2016/05/09/portland-jail-inmates-drug-death-classified-as-accidental/.

[CR35] Drug may be to blame for Albany County inmate’s death. (n.d.). WNYT. Retrieved May 11, 2020, from https://wnyt.com/news/grey-death-rare-drug-albany-county-jail/5189111

[CR36] Facing Addiction in America: The Surgeon General’s Spotlight on Opioids. (2019). National Intsitute of Health. https://www.drugabuse.gov/nidamed-medical-health-professionals/opioid-crisis-pain-management/facing-addiction-in-america-surgeon-generals-spotlight-opioids

[CR37] Fairbanks, P. (2016, September 9). Attica heroin and fentanyl overdoses lead to prison sentences. The Buffalo News. https://buffalonews.com/news/local/crime-and-courts/attica-heroin-and-fentanyl-overdoses-lead-to-prison-sentences/article_aef38c12-cff5-5a9e-9942-ccbb47b45bc3.html

[CR38] Fazel S, Benning R (2006). Natural deaths in male prisoners: A 20-year mortality study. European Journal of Public Health.

[CR39] Ferrise, A. (2019, June 3). Cuyahoga County releases video of jail overdose death that led to warden and officer’s indictment. Cleveland.Com. https://www.cleveland.com/metro/2019/06/cuyahoga-county-releases-video-of-jail-overdose-death-that-led-to-warden-and-officers-indictment.html

[CR40] Finn, L. (2020, June 9). *6 Inmates Overdose On Drugs in County Jails: Officials*. https://news.yahoo.com/6-inmates-overdose-drugs-county-225310068.html

[CR41] Gartrell, N. (2020, September 23). Man died of drug overdose in contra Costa jail, coroner finds. Mercury News. https://www.mercurynews.com/2020/09/23/man-died-of-drug-overdose-in-contra-costa-jail-days-after-he-was-arrested-and-briefly-hospitalized/

[CR42] Goldberg, T. (2018, May 1). Fentanyl blamed for apparent overdoses that killed California prisoner, sickened others | KQED. TQED. https://www.kqed.org/news/11665693/fentanyl-blamed-for-apparent-overdoses-that-killed-california-prisoner-sickened-others

[CR43] Goodrich, K. (2019, April 4). Blue Earth County Jail inmate allegedly overdosed on fentanyl. The Free Press. https://www.mankatofreepress.com/news/local_news/blue-earth-county-jail-inmate-allegedly-overdosed-on-fentanyl/article_13d04a58-5728-11e9-83c8-5334a7016a71.html

[CR44] Gottlieb, P. (2021, February 9). *Clallam County inmate overdoses while in jail*. https://www.sequimgazette.com/news/clallam-county-inmate-overdoses-while-in-jail/

[CR45] Green TC, Clarke J, Brinkley-Rubinstein L, Marshall BDL, Alexander-Scott N, Boss R, Rich JD (2018). Postincarceration fatal overdoses after implementing medications for addiction treatment in a statewide correctional system. JAMA Psychiatry.

[CR46] Green TC, Ray M, Bowman SE, McKenzie M, Rich JD (2014). Two cases of intranasal naloxone self-administration in opioid overdose. Substance Abuse.

[CR47] Harlow, D. (2017, May 5). Somerset County Jail inmate who overdosed saved by opioid antidote Narcan. Kennebec Journal and Morning Sentinel. https://www.centralmaine.com/2017/05/05/inmate-at-the-somerset-county-jail-who-overdosed-saved-by-opioid-antidote-narcan/

[CR48] Harris, J. (2019, December 3). *Autopsy: Columbia County inmates died after exposure to fentanyl*. News4jax. https://www.news4jax.com/news/2019/12/04/autopsy-columbia-county-inmates-died-after-exposure-to-fentanyl/

[CR49] Hlavaty, K. (2021, January 21). *Euclid man indicted for allegedly causing overdose death of cellmate in Cuyahoga County Jail*. https://www.news5cleveland.com/news/local-news/investigations/county-jail/euclid-man-indicted-for-allegedly-causing-overdose-death-of-cellmate-in-cuyahoga-county-jail

[CR50] Hopkins, K. (2017, November 1). 5 Alaska inmates overdosed within 24 hours at Hiland prison, officials say. Alaskas News Sourse. https://www.alaskasnewssource.com/content/news/5-Alaska-inmates-overdosed-within-24-hours-at-Hiland-prison-officials-say-454543013.html

[CR51] Horton M, McDonald R, Green TC, Nielsen S, Strang J, Degenhardt L, Larney S (2017). A mapping review of take-home naloxone for people released from correctional settings. International Journal of Drug Policy.

[CR52] *Increases in fentanyl drug confiscations and fentanyl-related overdose fatalities*. (2018). Center for Disease Control and Prevention. https://emergency.cdc.gov/han/han00384.asp

[CR53] Ireland, E. (2020, December 7). A drug Overdose killed vista jail inmate, medical examiner says—Times of San Diego. Times of San Diego. https://timesofsandiego.com/crime/2020/12/07/a-drug-overdose-killed-san-diego-jail-inmate-county-says/

[CR54] Ireland, E. (2021, January 13). A drug Overdose killed Otay Mesa jail inmate*,* Medical Examiner Says. Times of San Diego. https://timesofsandiego.com/crime/2021/01/12/a-drug-overdose-killed-otay-mesa-jail-inmate-medical-examiner-says/

[CR55] Iroquois County inmate died of fentanyl, heroin overdose. (2020, September 29). https://newschannel20.com/news/local/iroquois-county-inmate-died-of-fentanyl-heroin-overdose

[CR56] *Jail sweep underway after Pasco inmate dies from drug overdose*. (2019, February 1). [Text.Article]. FOX 13 Tampa Bay; FOX 13 Tampa Bay. https://www.fox13news.com/news/jail-sweep-underway-after-pasco-inmate-dies-from-drug-overdose

[CR57] Kemp, K. (n.d.). Deputy assisting inmate with drug Overdose fell ill yesterday in the Mendocino County jail. Redheaded Blackbelt. Retrieved April 12, 2021, from https://kymkemp.com/2020/12/09/deputy-assisting-inmate-with-drug-overdose-fell-ill-yesterday-in-the-mendocino-county-jail/

[CR58] Kim D, Irwin KS, Khoshnood K (2009). Expanded access to naloxone: Options for critical response to the epidemic of opioid overdose mortality. American Journal of Public Health.

[CR59] Kim S, Ting A, Puisis M, Rodriguez S, Benson R, Mennella C, Davis F (2007). Deaths in the Cook County jail: 10-year report, 1995–2004. Journal of Urban Health.

[CR60] Kinlock TW, Gordon MS, Schwartz RP, Fitzgerald TT, O’Grady KE (2009). A randomized clinical trial of methadone maintenance for prisoners: Results at 12 months postrelease. Journal of Substance Abuse Treatment.

[CR61] Kinlock TW, Gordon MS, Schwartz RP, O’Grady K, Fitzgerald TT, Wilson M (2007). A randomized clinical trial of methadone maintenance for prisoners: Results at 1-month post-release. Drug and Alcohol Dependence.

[CR62] Kinlock TW, Gordon MS, Schwartz RP, O’Grady KE (2008). A study of methadone maintenance for male prisoners: 3-month Postrelease outcomes. Criminal Justice and Behavior.

[CR63] Klein, D. (2018, December 2). Man sentenced for manslaughter in overdose death of inmate. WSAZ. https://www.wsaz.com/content/news/One-dead-several-treated-after-overdoses-at-Boyd-County-Jail-501733371.html

[CR64] Kobayashi L, Green TC, Bowman SE, Ray MC, McKenzie MS, Rich JD (2017). Patient simulation for assessment of layperson Management of Opioid Overdose with intranasal naloxone in a recently released prisoner cohort. Simulation in Healthcare: Journal of the Society for Simulation in Healthcare.

[CR65] Kolind T, Duke K (2016). Drugs in prisons: Exploring use, control, treatment and policy. Drugs: Education, Prevention and Policy.

[CR66] Korte, K. (2018, September 14). Cocaine laced with fentanyl leads to multiple deaths, overdoses. Drug Enforcement Administration. https://www.dea.gov/press-releases/2018/09/14/cocaine-laced-fentanyl-leads-multiple-deaths-overdoses

[CR67] Larney S, Farrell M (2017). Prisoner suicide: A multilevel problem. The Lancet. Psychiatry.

[CR68] Larney S, Gisev N, Farrell M, Dobbins T, Burns L, Gibson A (2014). Opioid substitution therapy as a strategy to reduce deaths in prison: Retrospective cohort study. BMJ Open.

[CR69] Larney S, Topp L, Indig D, O’Driscoll C, Greenberg D (2012). A cross-sectional survey of prevalence and correlates of suicidal ideation and suicide attempts among prisoners in New South Wales, Australia. BMC Public Health.

[CR70] Lee, C. (2020). *Investigation ordered into Mendocino County deputy’s suspected drug overdose*. https://www.pressdemocrat.com/article/news/investigation-ordered-into-mendocino-county-deputys-suspected-drug-overdos/

[CR71] Lisenby, A. L. O. S. L. P. (2018, December 27). Workhouse inmate died of opioid overdose. St. Louis American. http://www.stlamerican.com/news/local_news/workhouse-inmate-died-of-opioid-overdose/article_5ff1f39c-0961-11e9-b8d8-53d4cb7a0aff.html

[CR72] Loannou, F. (2017, July 20). Fentanyl suspected in inmate overdoses at Santa Clara County jail. SFGate. https://www.sfgate.com/bayarea/article/Fentanyl-suspected-in-inmate-overdoses-at-Santa-11303604.php

[CR73] Lubinski, A. (2020, December 21). 1 dead, others treated after possible fentanyl exposure at county jail. Courier-Tribune. https://www.mycouriertribune.com/news/1-dead-others-treated-after-possible-fentanyl-exposure-at-county-jail/article_93cbe7c2-7c1a-11ea-8186-07a289298bdd.html

[CR74] Mazhnaya A, Bojko MJ, Marcus R, Filippovych S, Islam Z, Dvoriak S, Altice FL (2016). In their own voices: Breaking the vicious cycle of addiction, treatment and criminal justice among people who inject drugs in Ukraine. Drugs: Education, Prevention and Policy.

[CR75] McDonald, J. (2020, February 6). Inmate charged in death of disbarred lawyer who died in custody last week. San Diego Union-Tribune. https://www.sandiegouniontribune.com/news/watchdog/story/2020-02-06/inmate-charged-in-death-of-disbarred-lawyer-who-died-in-custody-last-week

[CR76] McKelway, B. (2015, May 5). Suit seeks $10 million in Henrico inmate drug death. Richmond Times-Dispatch. https://richmond.com/news/local/henrico/suit-seeks-million-in-henrico-inmate-drug-death/article_f1d61723-f1a5-5799-9dff-f5e2eb6df4a1.html

[CR77] Michell. (2016, June 23). Parents of 25-year-old inmate who died in Gunnison jail of drug overdose file suit. The Denver Post. https://www.denverpost.com/2016/06/23/gunnison-jail-drug-overdose-lawsuit/

[CR78] Miller. (2018, November 21). Woman who hid drugs in vagina charged with murder after inmate ODs: Cops. The New York Post. https://nypost.com/2018/11/21/woman-who-hid-drugs-in-vagina-charged-with-murder-after-inmate-ods-cops/

[CR79] Minneapolis Man Charged With Smuggling Heroin Into Hennepin County Jail. (n.d.). Retrieved April 12, 2021, from https://www.cbsnews.com/live/cbsn-local-min/

[CR80] Mitchell, S. (2018, April 22). 5 Sumner inmates taken to hospital after suspected drug overdose. The Gallatin News. https://www.gallatinnews.com/news/crime/5-sumner-inmates-taken-to-hospital-after-suspected-drug-overdose/article_fb818d6e-4660-11e8-8aea-8fe1a876b584.html

[CR81] Mumola, C. J. (n.d.). *Suicide and Homicide in State Prisons and Local Jails* (p. 12). U.S. Department of Justice, Office of Justice Programs.

[CR82] Murphy, B. (n.d.). State: Inmate fatally overdosed after Nassau jail search failure. Newsday. Retrieved April 12, 2021, from https://www.newsday.com/long-island/crime/nassau-jail-death-drug-contraband-1.50132012

[CR83] Naylor, S. (2018, January 31). *Inmate at Cumberland County Prison died from fentanyl overdose, coroner says*. Fox43.Com. https://www.fox43.com/article/news/local/contests/inmate-at-cumberland-county-prison-died-from-fentanyl-overdose-coroner-says/521-ff4cd46b-9c11-4562-b38c-2e5f1ee861be

[CR84] Nelson, J. (2020, September 21). Charges: 3 inmates overdose after man smuggles heroin into jail. Bring Me The News. https://bringmethenews.com/minnesota-news/charges-3-inmates-overdose-after-minneapolis-man-smuggles-heroin-into-jail

[CR85] Nuttle, M. (2021, January 28). *4 Sacramento jail inmates revived after suspected overdoses*. Abc10.Com. https://www.abc10.com/article/news/crime/4-inmates-at-sacramento-county-jail-revived-after-suspected-drug-overdoses/103-fa3ac6a3-e3d3-4ccd-8ab9-82ea9c174605

[CR86] O’Donnell JK, Halpin J, Mattson C, Goldberger B, Gladden M (2017). Deaths involving fentanyl, fentanyl analogs, and U-47700—10 states, July–December 2016. MMWR. Morbidity and Mortality Weekly Report.

[CR87] OC Inmate’s Death Caused By Fentanyl Overdose, Officials Say. (2020, April 18). https://patch.com/california/orange-county/oc-inmates-death-caused-fentanyl-overdose-officials-say

[CR88] Ostling PS, Davidson KS, Anyama BO, Helander EM, Wyche MQ, Kaye AD (2018). America’s opioid epidemic: A comprehensive review and look into the rising crisis. Current Pain and Headache Reports.

[CR89] Ovalle, D. (2017, December 7). *Two Miami jail inmates dead from possible fentanyl overdoses | Miami Herald*. https://www.miamiherald.com/news/local/crime/article188576814.html

[CR90] Owens, D. (2019, April 9). Inmate who overdosed on fentanyl at Hartford Correctional Center dies. Hartford Courant. https://www.courant.com/news/connecticut/hc-hartford-correctional-overdose-death-0410-20190409-ua4b5sijybhspff4fdy3fm5tvm-story.html

[CR91] Parmar MKB, Strang J, Choo L, Meade AM, Bird SM (2017). Randomized controlled pilot trial of naloxone-on-release to prevent post-prison opioid overdose deaths. Addiction (Abingdon, England).

[CR92] *Pasco County man responsible for numerous overdoses at Pasco County jail sentenced to 27 years in federal prison*. (2020, March 12). Drug Enforcement Administration. https://www.dea.gov/press-releases/2020/03/12/pasco-county-man-responsible-numerous-overdoses-pasco-county-jail

[CR93] *Pasco Man Pleads Guilty To Causing Series Of Overdoses While Incarcerated At The Pasco County Jail*. (2019, November 4). The United States Department of Justice, U.S. Attorney’s Office. https://www.justice.gov/usao-mdfl/pr/pasco-man-pleads-guilty-causing-series-overdoses-while-incarcerated-pasco-county-jail

[CR94] Patrick, Robert. (2019, May 23). *Inmate dies in St. Charles County jail | Law and order |* stltoday.com. St. Lousi today. https://www.stltoday.com/news/local/crime-and-courts/inmate-dies-in-st-charles-county-jail/article_7624df6d-c862-55d6-ac94-19d8e353be40.html

[CR95] Patrick, Roberts. (2020, July 8). Family of inmate sues over his overdose death in St. Charles County jail | law and order | stltoday.com. https://www.stltoday.com/news/local/crime-and-courts/family-of-inmate-sues-over-his-overdose-death-in-st-charles-county-jail/article_da556bc9-8ae8-57a7-8f99-8989424ad256.html

[CR96] Perlstein, M. (2019, December 3). Fentanyl overdose in Orleans parish jail not an isolated incident, sources say. Wwltv.Com. https://www.wwltv.com/article/news/investigations/family-sues-after-father-overdoses-on-fentanyl-in-new-orleans-jail/289-008b5cee-0f60-4d0a-98d9-031bcac003bf

[CR97] Petterson AG, Madah-Amiri D (2017). Overdose prevention training with naloxone distribution in a prison in Oslo, Norway: A preliminary study. Harm Reduction Journal.

[CR98] Poli, D. (n.d.). Vermont man dies at greenfield jail. In *Greenfield recorder* Retrieved April 12, 2021, from https://www.recorder.com/Vermont-man-dies-at-Massachusetts-jail-29414844.

[CR99] Prison Opioid Project – Medication for Opioid Use Disorder and the Criminal Justice System. (n.d.). Retrieved April 19, 2021, from http://dev.prisonopioidproject.org/

[CR100] Rains, B. (2019, March 1). *Fentanyl blamed for Sebastian County inmate’s death*. 4029 news. https://www.4029tv.com/article/fentanyl-blamed-for-sebastian-county-inmates-death/26596683

[CR101] *Reported Law Enforcement Encounters Testing Positive for Fentanyl Increase Across US | Drug Overdose | CDC Injury Center*. (2019, August 20). Center for Disease Control and Prevention. https://www.cdc.gov/drugoverdose/data/fentanyl-le-reports.html

[CR102] Rich, J. D., McKenzie, M., Larney, S., Wong, J. B., Tran, L., Clarke, J., Noska, A., Reddy, M., & Zaller, N. (2015). Methadone continuation versus forced withdrawal on incarceration in a combined US prison and jail: A randomised, open-label trial. Lancet (London, England), 386(9991), 350–359. 10.1016/S0140-6736(14)62338-2 10.1016/S0140-6736(14)62338-2 PMC4522212 26028120

[CR103] Riggins, A. (2021, January 13). Authorities say man who died at Otay Mesa jail overdosed on fentanyl. San Diego Union-Tribune. https://www.sandiegouniontribune.com/news/public-safety/story/2021-01-13/authorities-say-man-who-died-at-otay-mesa-jail-overdosed-on-fentanyl

[CR104] Rogers, Dave. (2019, September 11). Coroner*:* Middleton Jail inmate died of drug overdose. https://www.newburyportnews.com/news/local_news/coroner-middleton-jail-inmate-died-of-drug-overdose/article_861909ce-0bf7-509f-89e3-eff658a81a56.html

[CR105] Rogers, Doug. (2015, May 21). Inmate Found Guilty on All Charges in Prison Overdoses. Erie News Now. https://www.erienewsnow.com/story/29125008/inmate-found-guilty-on-all-charges-in-prison-overdoses

[CR106] Saavedra, T. (2020). *Use of naloxone to combat opioid overdoses soars in Orange County jails – Orange County Register*. https://www.ocregister.com/2020/01/15/use-of-naloxone-to-combat-opioid-overdoses-soars-in-orange-county-jails/

[CR107] Salive ME, Smith GS, Brewer TF (1990). Death in prison: Changing mortality patterns among male prisoners in Maryland, 1979-87. American Journal of Public Health.

[CR108] Sandy, E. (2016, April 21). Fentanyl Overdose Cited in Lorain County Jail Inmate’s Death. Cleveland Scene. https://www.clevescene.com/scene-and-heard/archives/2016/04/21/fentanyl-overdose-blamed-for-lorain-county-jail-inmates-death

[CR109] Saunders, M. (2020, December 12). San Diego deputies treat 6 inmates for potential fentanyl overdose. KGTV. https://www.10news.com/news/local-news/san-diego-deputies-treat-6-inmates-for-potential-fentanyl-overdose

[CR110] Schaefer, C. (2016, December 19). Coroner*: Dustin Rybak died of fentanyl overdose in jail*. WRGT. https://dayton247now.com/news/local/coroner-dustin-rybak-died-of-fentanyl-overdose-in-jail

[CR111] Schafer, A. (2020, December 2). Manitowoc County Jail death of Justin Hall caused by fentanyl overdose. Herald Times Reporter. https://www.htrnews.com/story/news/2020/12/02/manitowoc-county-jail-death-justin-hall-caused-fentanyl-overdose/3795633001/

[CR112] Schmidt, S. J. (2020, February 7). Woman who smuggled fentanyl into Madison County jail, leading to overdoses, gets 11-years. Alton Telegraph. https://www.thetelegraph.com/news/article/Woman-gets-11-years-for-drugs-brought-into-jail-15039554.php

[CR113] Schwigert, K. (2019, April 24). Two Lancaster men charged in connection to Lancaster County Prison inmate’s fatal fentanyl overdose. Vox. https://www.fox43.com/article/news/local/contests/two-lancaster-men-charged-in-connection-to-lancaster-county-prison-inmates-fatal-fentanyl-overdose/521-a9997561-6233-43d9-9dc0-7203007386b7

[CR114] Sheriff’s Office Conducts Drug Search in Jail After Drug Overdose. (n.d.). Retrieved April 12, 2021, from https://bladenonline.com/sheriffs-office-conducts-drug-search-in-jail-after-drug-overdose/

[CR115] Signorini, R. (2019, May 17). *Allegheny County Jail inmate found dead in cell died of drug overdose |* TribLIVE.com. TribLIVE. https://triblive.com/local/pittsburgh-allegheny/medical-examiner-jail-inmate-died-from-drug-overdose/

[CR116] Sledge, M. (2020, October 18). Fentanyl, veterinary drug were in body of New Orleans jail inmate who overdosed, coroner says. NOLA.Com. https://www.nola.com/article_9433131c-0ffd-11eb-ab3c-3bf4ba8d4d1e.html

[CR117] Smay, I. (2019, July 30). *Inmate who died Spokane Co. Jail in June overdosed, medical examiner says*. Krem2. https://www.krem.com/article/news/local/spokane-county/inmate-who-died-spokane-co-jail-in-june-overdosed-medical-examiner-says/293-8db03c03-6d92-4c0a-afd7-8bb9e7be9a31

[CR118] Stanton, S. (2021, January 28). Suspected overdoses at Sacramento County Main Jail send inmates to area hospital. The Sacramento Bee. https://www.sacbee.com/news/local/article248857584.html

[CR119] Stucker, K. (2019, January 11). Woman sentenced in fatal jailhouse overdose. Fosters.Com. https://www.fosters.com/news/20190111/woman-sentenced-in-fatal-jailhouse-overdose

[CR120] Sturgeon, C. (2015, July 14). Three inmates overdose on heroin at York County Jail. WMTW. https://www.wmtw.com/article/three-inmates-overdose-on-heroin-at-york-county-jail/2009554

[CR121] Sutyak, K. (2018, December 10). Inmate at Portage County jail treated for possible fentanyl overdose; nurse given Narcan after exposure to drugs. *Fox 8 Cleveland WJW*. https://fox8.com/news/inmate-at-portage-county-jail-treated-for-possible-fentanyl-overdose-nurse-given-narcan-after-exposure-to-drugs/

[CR122] Suzuki J, El-Haddad S (2017). A review: Fentanyl and non-pharmaceutical fentanyls. Drug and Alcohol Dependence.

[CR123] Treleven, E. (2020, June 8). Estate of jail inmate sues over 2016 drug overdose death. Wisconson State Journal. https://madison.com/wsj/news/local/crime-and-courts/estate-of-jail-inmate-sues-over-2016-drug-overdose-death/article_8861c117-9e02-5381-ae89-1cfeaeb83a79.html

[CR124] *U.S. drug overdose deaths continue to rise; increase fueled by synthetic opioids*. (2019). The Center for Disease Control and Prevention. https://www.cdc.gov/media/releases/2018/p0329-drug-overdose-deaths.html

[CR125] USDA ERS - Rural-Urban Continuum Codes. (n.d.). Retrieved April 12, 2021, from https://www.ers.usda.gov/data-products/rural-urban-continuum-codes.aspx

[CR126] Ward, K. (n.d.). If an inmate dies of an overdose, who is to blame? This lawsuit says jailers are*.* Lexington Herald-Leader. Retrieved May 11, 2020, from https://www.kentucky.com/news/local/crime/article211944594.html

[CR127] WDTV News Staff. (2020, December 6). *2 inmates dead of suspected overdose; 1 held less than a day*. https://www.wdtv.com/2020/12/06/overdose-suspected-in-deaths-of-2-inmates-at-wva-jail/

[CR128] Wenger LD, Showalter D, Lambdin B, Leiva D, Wheeler E, Davidson PJ (2019). Overdose education and naloxone distribution in the San Francisco County jail. Journal of Correctional Health Care.

[CR129] Wester, J. (2019, June 10). Mecklenburg County jail imate overdosed on fentanyl inside the jail, autopsy says*.* Charlotte Observer. https://www.charlotteobserver.com/news/local/crime/article231383653.html

[CR130] Wester, Jane. (n.d.). *Another jail inmate died of fentanyl overdoses this summer, records show*. The Charlotte observer. Retrieved April 12, 2021, from https://www.charlotteobserver.com/news/local/crime/article222946255.html

[CR131] Wester, Jane, & Kane, D. (2018, November 6). Autopsies show drug overdoses in two jail deaths. Charlotte Observer. https://www.charlotteobserver.com/news/local/crime/article221512330.html

[CR132] Winkelman TNA, Chang VW, Binswanger IA (2018). Health, Polysubstance use, and criminal justice involvement among adults with varying levels of opioid use. JAMA Network Open.

[CR133] Winston, H. (2020, July 11). Inmate in PBC jail for six days dies of fentanyl overdose*, ME says*. https://www.palmbeachpost.com/story/news/courts/2020/06/11/inmate-in-pbc-jail-for-six-days-dies-of-fentanyl-overdose-me-says/41746543/

[CR134] WKBN Staff. (2019, March 22). Deputies say inmate overdosed on fentanyl at Mahoning County jail. *WKBN.Com*. https://www.wkbn.com/news/local-news/deputies-say-inmate-overdosed-on-fentanyl-at-mahoning-county-jail/

[CR135] *Woman died of fentanyl overdose at Cook County Jail: Autopsy*. (2019, March 28). [Text.Article]. FOX 32 Chicago; FOX 32 Chicago. https://www.fox32chicago.com/news/woman-died-of-fentanyl-overdose-at-cook-county-jail-autopsy

